# Intraprocedural 3D-vena contracta area predicts survival after transcatheter edge-to-edge repair: results from MITRA-PRO registry

**DOI:** 10.1007/s00392-024-02580-6

**Published:** 2024-12-09

**Authors:** Dennis Rottländer, Jörg Hausleiter, Thomas Schmitz, Alexander Bufe, Melchior Seyfarth, Ralph Stephan von Bardeleben, Harald Beucher, Taoufik Ouarrak, Steffen Schneider, Peter Boekstegers, the MITRA-PRO investigators

**Affiliations:** 1 Department of Cardiology, Faculty of Health, School of Medicine, Witten, Witten/Herdecke University, Alfred-Herrhausen-Straße 50, 58455 Witten, Germany; 2 Department of Cardiology, Krankenhaus Porz Am Rhein, Cologne, Germany; 3 Department of Cardiology, Klinikum Der Universität München, Ludwig-Maximilians-Universität, Munich, Germany; 4 DZHK (German Center for Cardiovascular Research), Partner Site Munich Heart Alliance, Munich, Germany; 5 Department of Cardiology, Elisabeth Krankenhaus, Essen, Germany; 6 Department of Cardiology, Heart Centre Niederrhein, Helios Clinic Krefeld, Krefeld, Germany; 7 Department of Cardiology, University Hospital Helios Wuppertal, Wuppertal, Germany; 8 Department of Cardiology, University Medical Center of Mainz, Mainz, Germany; 9 Department of Cardiology, Helios Klinikum Siegburg, Siegburg, Germany; 10 Institut Für Herz-Infarkt Forschung, Ludwigshafen, Germany

**Keywords:** Transcatheter mitral valve repair, Mitral regurgitation, 3D vena contracta area, Edge-to-edge repair, Residual regurgitation

## Abstract

**Background:**

The MITRA-PRO registry revealed residual mitral regurgitation (MR) to be an important predictor of survival following transcatheter edge-to-edge repair (TEER). Intraprocedural MR assessment using 3D-Vena Contracta Area (VCA) might be a feasible tool to guide mitral TEER procedures. The study aimed to assess the impact of residual MR assessed by 3D-VCA on 1-year mortality.

**Methods:**

823 patients with residual MR quantification using 3D-VCA in the MITRA-PRO registry, were included in this study. 1-year mortality, NYHA classification and major adverse events were assessed 1-year after mitral TEER.

**Results:**

Patients with trace residual MR after mitral TEER were allocated to the 3D-VCA < 0.1 cm^2^ group (27.8%), while a 3D-VCA ≥ 0.1 < 0.3 cm^2^ (55.4%) was considered as mild and a 3D-VCA ≥ 0.3 cm^2^ (16.8%) as relevant residual MR. One-year mortality was significantly lower in patients with non-relevant residual MR (3D-VCA < 0.1 cm^2^: 10.5%; ≥ 0.1 < 0.3 cm^2^: 16.0%; ≥ 0.3: 24.8%, p = 0.003). An increasing 3D-VCA post mitral TEER was associated with a higher 1-year mortality. At a 3D-VCA of 0.07 cm^2^ mortality increased significantly (1-year mortality 3D-VCA post mitral TEER ≥ 0.07 cm^2^: 16.5% vs. < 0.07 cm^2^: 7.8%; p = 0.005) indicating a 3D-VCA of 0.07 cm^2^ to be a cut-off value for survival in daily practice.

**Conclusions:**

Residual MR assessed by 3D-VCA after TEER is associated with 1-year mortality. Therefore, 3D-VCA is a valuable echocardiographic tool for intraprocedural MR assessment during mitral TEER and achieving a lower 3D-VCA improve patient survival. (German Clinical Trials Register: DRKS00012288).

**Trial Registration Number:**

DRKS00012288

**Supplementary Information:**

The online version contains supplementary material available at 10.1007/s00392-024-02580-6.

## Introduction

The recently published results of the MITRA-PRO registry confirmed that residual mitral regurgitation (MR) is associated with 1-year mortality and rehospitalization in patients with functional, degenerative or mixed MR undergoing mitral transcatheter edge-to-edge repair (TEER) [1]. Assessment of MR severity via echocardiographic eyeballing is often used to determine residual regurgitation, while quantitative echocardiographic parameters like 3D-VCA are not routinely used during TEER. However, 3D-VCA is an emerging parameter for residual MR quantification following TEER [2–4]. One might speculate, that including 3D-VCA in clinical routine of intraprocedural residual MR assessment might be beneficial for prognosis assessment and verification of procedural results. 3D-VCA correlates with hemodynamic parameters and is reduced after mitral TEER [3]. In a small cohort of patients, a 3D-VCA threshold of 0.27 cm^2^ was recently identified to indicate at least moderate MR with good diagnostic accuracy and a negative predictive value of 92% [3]. Also, a smaller 3D-VCA was associated with an improvement of NYHA functional class at 30-day follow-up [3]. Therefore, measurement of 3D-VCA during intraprocedural transesophageal echocardiography (TOE) might be a feasible tool for interventional cardiologists to guide mitral TEER procedures. It might provide reliable quantification of residual MR following TEER using a single parameter. However, no validated grading of 3D-VCA exists and current guidelines do not recommend 3D-VCA for residual MR graduation following TEER [5, 6]. Furthermore, applicable studies are most often retrospective, single-center and include only small sample sizes. Validated cut-off values for residual MR grading following TEER are still missing and no correlation of these cut-off values to mortality exists. We aimed to evaluate, whether intraprocedural 3D-VCA assessment of residual MR alone is able to predict prognosis following mitral TEER.

## Methods

### Study population

**Fig. 1:**
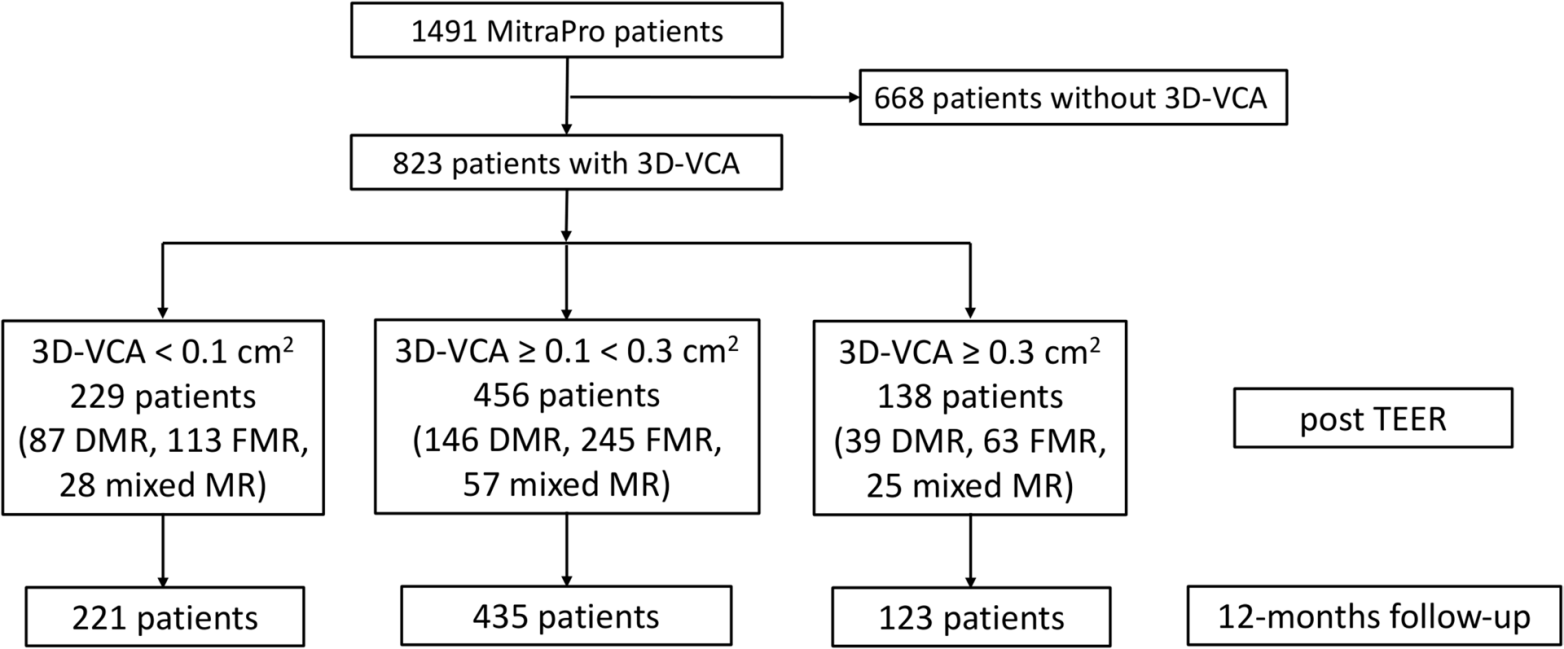
Study flow chart. *TEER* transcatheter edge-to-edge repair, *3D-VCA* 3D-Vena Contracta Area, *DMR* degenerative mitral regurgitation, *FMR* functional mitral regurgitation; *mixed MR* mixed mitral regurgitation

The prospective, multicenter MITRA-PRO (MITRAclip PROgnosis) registry includes 24 centers in Germany (German Clinical Trials Register: DRKS00012288). All participating study centers were recently published [1]. The registry was designed to evaluate the association of residual intraprocedural MR following TEER on 1-year mortality and rehospitalization for heart failure. The MITRA-PRO registry was performed according to good clinical practice and in compliance with the Helsinki declaration. An individual written consent was obtained from every patient. The study was approved by the ethical committee of the University of Witten-Herdecke (approval number: 96/2016) and the local ethic committees of the 24 centers, if appropriate. The MITRA-PRO registry database contains 1546 patients as previously published [1]. 3D-VCA was assessed in 823 patients prior and post mitral TEER. It was part of the MitraScore calculation, depending on the investigator's decision [1]. Clinical follow-ups were done investigator independent by a central site (ZKS Witten/Herdecke University), while 12-month transthoracic echocardiography was performed by the individual study centers. Patients with a trace residual MR after mitral TEER were allocated to the 3D-VCA < 0.1 cm^2^ group (0.07 cm^2^ defined the cut-off for increased mortality in a ROC analysis and was rounded to 0.1 cm^2^), while a 3D-VCA ≥ 0.1 < 0.3 cm^2^ was considered as mild and a 3D-VCA ≥ 0.3 cm^2^ as a relevant residual MR (0.27 cm^2^ rounded to 0.3 cm^2^ as previously published [3]). 2D-TOE analysis by an independent corelab was performed in 292 patients, while 47 patients had 3D-VCA raw data pre and post mitral TEER available. Figure 1 shows the study flow chart.

### Mitral TEER

Mitral TEER was performed using either MitraClip XT, NT, NTR or XTR (Abbott Structural Heart, Santa Clara, California) as previously published [7]. The procedure was guided by TOE. The study protocol for MR quantification included a detailed approach for MR assessment. This involved the investigator's visual estimation, pulmonary vein flow, color flow jet area, jet density, jet contour, and vena contracta. Proximal isovelocity surface area (PISA), effective regurgitant orifice area (EROA), and regurgitant volume were used only when appropriate and were excluded from MR quantification after mitral TEER.

### 3D vena contracta area

**Fig. 2:**
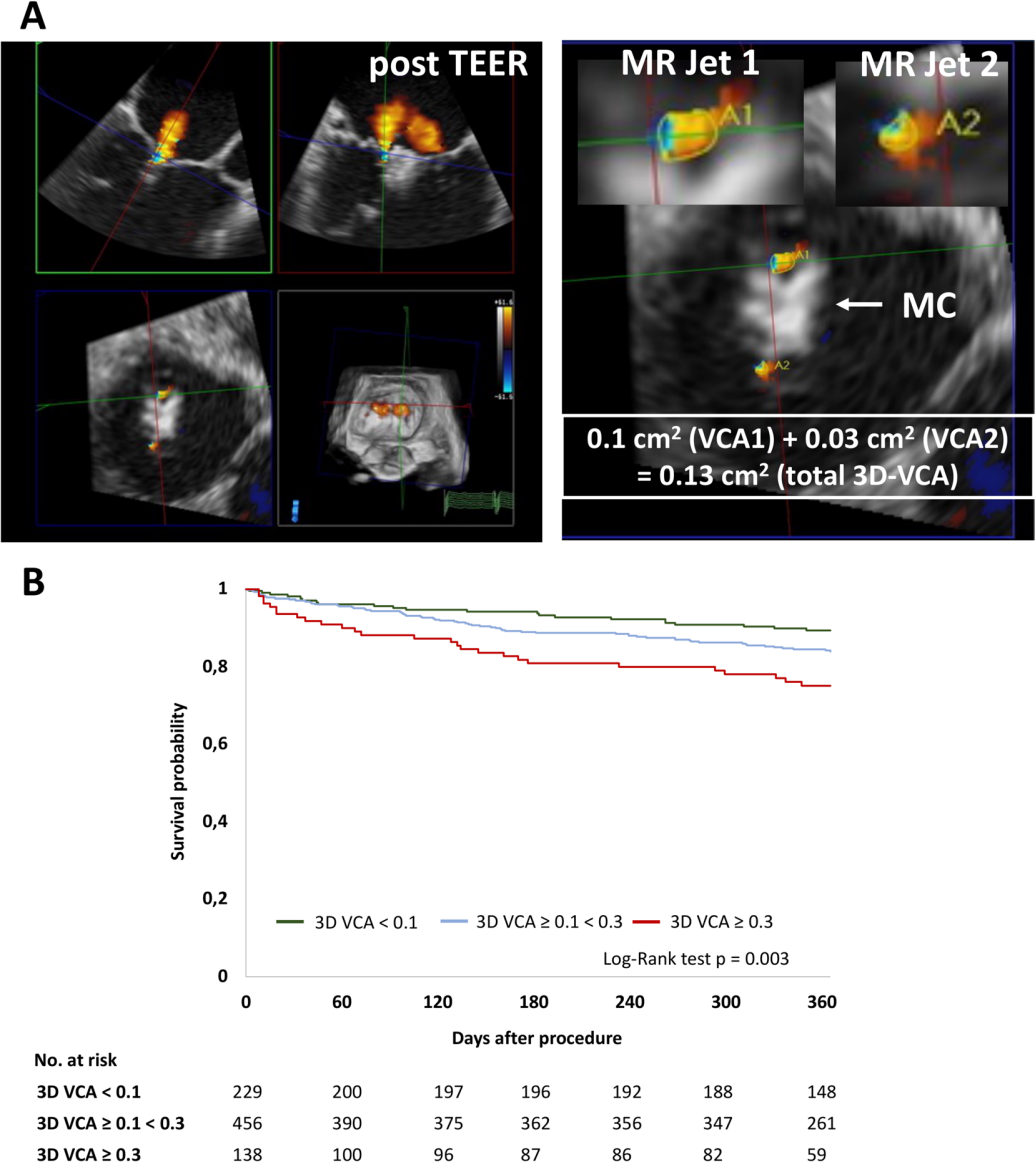
Intraprocedural 3D-VCA assessment post mitral TEER and long-term survival. (**A**) Summation method for 3D-VCA assessment post mitral transcatheter edge-to-edge repair (TEER). In case of more than 1 residual regurgitant jet, each jet was assessed singularly, its orthogonal planes identified, and the total VCA is calculated as the sum of all individual 3D-VCAs. *MR* mitral regurgitation, *MC* MitraClip. (**B**) Kaplan–Meier curve for 1-year mortality in patients with 3D-VCA < 0.1 cm^2^ (green line), ≥ 0.1 < 0.3 cm^2^ (blue line) or ≥ 0.3 cm^2^ (red line). 3D-VCA assessed post TEER

According to the study protocol all mandatory MR TOE measurements were performed at baseline after guide insertion to the left atrium and 10 min after deployment of the final TEER device (before retraction of the guiding catheter into the right atrium). In 823 patients 3D-VCA was available prior and post mitral TEER. 3D-VCA evaluation was performed from the investigators in a real-world setting at the corresponding study site. 3D color Doppler imaging of the mitral valve was performed from mid-esophageal TOE views. Six ECG triggered sequential 3D–scans were mandatory for a 3D–color full volume dataset. The software used for 3D-VCA analysis was depended on the study center. Supplementary Table 1 shows a list of the study centers including the number of 3D-VCA patients, the echocardiography system used and the software for 3D-VCA analysis. All recorded 3D–color full volume datasets were checked by the investigators for stitching artefacts, blooming effects or “dropouts” within the dataset. In case of artefacts patients were excluded from 3D-VCA measurements. The 3D frame rate was recommended to be around 15–25 Hz, as recommended at the timepoint of study initiation [8]. To avoid stitching artefacts in patients with atrial fibrillation a lower number of subvolumes was used or a 3D color Doppler loop with high volume rate was acquired. Furthermore, in patients with atrial fibrillation during the procedure 3D-VCA was calculated as mean of a minimum of 3–5 measurements. 3D-VCA was defined as the cross-sectional area of the narrowest portion of the proximal regurgitant jet through the closed mitral valve in systole [9–11]. 3D-volumes were manually cropped by the investigators to provide a direct view perpendicular to the jet direction. For 3D-VCA quantification, the 3D dataset was rotated to bisect the long axis of the jet in each of the two orthogonal planes. The third plane (short axis) was then scanned from proximal to distal in order to identify the narrowest portion of the jet immediately distal to the regurgitant orifice. Afterwards manual planimetry of the color Doppler signal was performed. Dark or very light color signals, which are of very low or high velocities were excluded of the MR jet (which is typically 400–600 cm/s). In case of multiple residual MR jets, 3D-VCA of each jet was determined separately and added up (Fig. 2).

In 47 patients 3D-VCA pre and post mitral TEER was measured by the investigator during the index procedure as well as an independent core-lab. The core lab was blinded to the clinical patient data and the intraprocedural results. Both, investigator and core lab were blinded to each other and an independent statistician performed interobserver variability analysis.

### Intraprocedural hemodynamics

All investigators minimized MR by performing TEER without exceeding the threshold of 5 mmHg mean pressure gradient across the mitral valve determined by continuous wave Doppler. All hemodynamic MR measurements were performed at baseline after guide insertion and 10 min after deployment of the last TEER device. Peri-procedural fluid administration was monitored by left ventricular end-diastolic pressure (LVEDP) or central venous pressure (CVP) aiming for an LVEDP greater than 10 mmHg and a central venous pressure between 10 and 15 mmHg. Systolic arterial blood pressure was adjusted to the patient´s preprocedural level by norepinephrine and/or fluid substitution. Left atrial pressure (LAP) was assessed prior and post mitral TEER.

### Statistical analysis

Categorical parameters are described using absolute numbers and percentages and were compared using the Pearson chi-square test, continuous variables were presented as mean and standard deviation or median and quartiles and were compared using the Kruskal–Wallis test. A logistic regression model was used to identify predictors of 3D-VCA ≥ 0.3 cm^2^ after TEER at baseline. In addition to age and gender, the following baseline characteristics with *p* < 0.1 from the univariate analysis (3D-VCA ≥ 0.3 cm^2^ vs. < 0.3 cm^2^) were included in the model: sPAP per 20 mmHg, permanent atrial fibrillation, cardiac resynchronization therapy (CRT), mixed disease MR and non-ischemic cardiomyopathy. The cumulative incidence of mortality in the 3D-VCA groups was estimated using the Product-Limit method and were visualized as Kaplan–Meier curves with patients at risk and compared using log-rank test. Distribution of NYHA classes at baseline, discharge and 12-months follow-up according to the 3D-VCA groups was represented as bar chart. The impact of residual MR by 3D-VCA on the 1-year mortality was tested in an age- and sex adjusted logistic regression analyses and corresponding OR with 95%-CI were presented. The group 3D-VCA < 0.1 cm^2^ was used as reference. Interobserver variability of 3D-VCA (investigators versus core lab) was visualized using a Bland-Altmann-Plot. The plot provides a visual representation of the difference between two measurements on the y-axis and the average of the two measurements on the x-axis. The linear association between the 3D-VCA measurements (investigator versus core lab) was made with the Pearson correlation coefficient. The diagnostic values of echocardiographic parameters for MR quantification were analyzed by receiver operating characteristic (ROC) curve. All analyses were performed using SAS release 9.4 (Statistical Analysis Software, Cary, NC).

## Results

The MITRA-PRO registry enrolled 1491 consecutive individuals with primary, secondary, or mixed MR who underwent mitral TEER and intraprocedural echocardiographic assessment of residual MR. In a real-world scenario, in 823 patients 3D-VCA before and after mitral TEER were part of the MitraScore calculations (Fig. 1). Following a ROC analysis at a 3D-VCA of 0.07 cm^2^, mortality significantly increased (1-year mortality for 3D-VCA post-mitral TEER ≥ 0.07 cm^2^: 16.5% vs. < 0.07 cm^2^: 7.8%; p = 0.005), highlighting a 3D-VCA value of rounded 0.1 cm^2^ as a relevant cutoff for survival assessment. Furthermore, a 3D-VCA of 0.27 cm^2^ was previously described as moderate residual MR (3). Therefore, we allocated the patients to 3 groups: 229 (27.8%) with a 3D-VCA of < 0.1 cm^2^, 456 (55.4%) with a 3D-VCA ≥ 0.1 < 0.3 cm^2^, and 138 (16.8%) with a 3D-VCA of ≥ 0.3 cm^2^. Group assignment was based on the 3D-VCA measurement post-mitral TEER.

### Baseline and procedural characteristics

The baseline characteristics of the 3D-VCA groups are shown in Table 1 and were similar, except a higher systolic pulmonary artery pressure (sPAP) in the 3D-VCA ≥ 0.3 cm^2^ group. Furthermore, a greater number of TEER devices were used, and the procedure time was significantly longer in patients with a 3D-VCA ≥ 0.3 cm^2^ post-mitral TEER (supplementary Table 2). Both CVP and LAP were elevated before and after mitral TEER in the 3D-VCA ≥ 0.3 cm^2^ group compared to the 3D-VCA < 0.1 cm^2^ or 3D-VCA ≥ 0.1 < 0.3 cm^2^ groups (supplementary Table 2).

**Table 1:** Patients characteristics at baseline

	3D VCA < 0.1 cm^2^ n = 229		3D VCA ≥ 0.1 < 0.3 cm^2^ n = 456		3D VCA ≥ 0.3 cm^2^ n = 138		p-value
	n	% or Mean ± SD or Median (Min,Max)	n	% or Mean ± SD or Median (Min,Max)	n	% or Mean ± SD or Median (Min,Max)	
Age	229	78.0 ± 7.0	456	78.0 ± 8.0	138	78.0 ± 8.0	0.90
Female	86	37.6	199	43.6	46	33.3	0.06
Log EuroScore (%)	214	23.3 ± 14.1	437	21.6 ± 14.4	127	22.2 ± 13.2	0.49
*Patients’ history*							
Arterial hypertension	203	88.6	396	86.3	120	87.6	0.80
Diabetes mellitus	56	24.5	131	28.7	34	24.8	0.41
Coronary artery disease	161	70.3	272	59.6	73	53.3	0.018
Previous TAVR	15	6.6	34	7.5	10	7.3	0.91
Previous CABG	44	19.2	75	16.4	27	19.7	0.54
Ischemic cardiomyopathy	75	32.8	151	33.1	43	31.4	0.93
Non-ischemic cardiomyopathy	49	21.4	107	23.5	37	27.0	0.47
Pacemaker	45	19.7	109	23.9	30	21.9	0.45
ICD	35	15.3	94	20.6	28	20.4	0.22
CRT	28	12.2	59	12.9	26	19.0	0.15
Atrial fibrillation	154	67.2	314	68.9	102	74.5	0.33
Paroxysmal	49	31.8	87	27.7	26	25.5	
Persistent	47	30.5	84	26.8	21	20.6	
Permanent	58	37.7	143	45.5	55	53.9	
TR mild	87	38.0	175	38.4	55	40.1	0.43
TR moderate	86	37.6	157	34.4	42	30.7	
TR severe	38	16.6	100	21.9	32	23.4	
RV dysfunction	103	45.0	198	43.4	61	44.5	0.92
TAPSE < 20 mm	74	33.0	173	38.2	55	40.1	0.31
*Echocardiography*							
only primary MR	87	38.0	146	32.0	39	28.3	0.12
only secondary MR Mixed disease MR	113 28	49.3 12.2	245 57	53.7 12.5	63 25	45.7 18.1	0.20 0.20
LVEF (%)	229	42.6 ± 14.3	453	44.7 ± 15.3	83	44.6 ± 17.4	0.17
sPAP (mmHg)	225	45.0 ± 13.6	441	48.8 ± 20.5	131	51.9 ± 16.2	< 0.001
*Dyspnea*							
NYHA I	0	0.0	3	0.7	0	0.0	0.74
NYHA II	19	8.3	34	7.5	14	10.2	
NYHA III	185	80.8	365	80.2	104	75.9	
NYHA IV	25	10.9	53	11.6	19	13.9	
*Laboratory results*							
NT-proBNP (pg/ml)	229	2744 (1268, 6032)	456	3309 (1541, 6414)	138	2820 (1833, 6260)	0.41
*Baseline medication*							
Beta blocker	200	87.3	392	86.0	114	83.2	0.55
ACEI or ARB or ARNI	166	72.5	325	71.3	99	72.3	0.94
MRA	81	35.4	162	35.5	48	35.0	0.99
Diuretic	206	90.0	427	93.6	126	92.0	0.23
Phenprocoumon	58	25.3	120	26.3	42	30.7	0.51
DOAK	98	42.8	175	38.4	59	43.1	0.42
Aspirin	79	34.5	146	32.0	34	24.8	0.15
Clopidogrel	63	27.5	102	22.4	34	24.8	0.33

*ICD* implantable cardioverter-defibrillator, *CRT* cardiac resynchronization therapy, *LVEF* left ventricular ejection fraction, *sPAP* systolic pulmonary artery pressure, *MR* mitral regurgitation, *TR* tricuspid regurgitation, *NYHA* New York Heart Association, *ACEI* angiotensin-converting enzyme inhibitor, *ARB* angiotensin receptor blocker, *ARNI* angiotensin receptor neprilysin inhibitor, *MRA* mineralocorticoid receptor antagonist, *DOAK* direct oral anticoagulant, *SD* standard deviation, *TAVR* transcatheter aortic valve replacement, *CABG* coronary artery bypass grafting

### Peri-interventional complications and in-hospital outcomes

Peri-interventional complications were rare across all groups, with no significant differences observed, as detailed in supplementary Table 2. During the hospital stay following the index procedure, there were 3 deaths in the 3D-VCA < 0.1 cm^2^ group, 9 deaths in the 3D-VCA ≥ 0.1 < 0.3 cm^2^ group, and 4 deaths in the 3D-VCA ≥ 0.3 cm^2^ group, resulting in in-hospital mortality rates of 1.3%, 2.0%, and 2.9%, respectively (p = 0.56; supplementary Table 2). Additionally, the rates of in-hospital major adverse cardiovascular events (MACE) and major adverse cardiovascular and cerebrovascular events (MACCE) were similar across all groups (supplementary Table 2).

### Predictors of 3D-VCA ≥ 0.3 cm^***2***^

To identify predictors of residual MR a multivariate analysis was performed. It revealed sPAP (per 20 mmHg), severe baseline MR and permanent AF as independent predictors of a 3D-VCA ≥ 0.3 cm^2^ (Table 2).

**Table 2:** Multivariable predictors of 3D VCA ≥ 0.3 cm^2^ after TEER at baseline

Odds ratio estimates and wald confidence intervals				
Effect	p-value	Estimate	95% Confidence Limits	
Gender (female)	0.1373	0.730	0.482	1.106
Age (per year)	0.8526	1.002	0.977	1.029
sPAP per 20 mmHg	0.0344*	1.243	1.016	1.521
Severe MR in baseline TOE	0.0148*	2.904	1.232	6.847
permanent AF	0.0366*	1.533	1.027	2.289
CRT	0.1872	1.437	0.839	2.461
Mixed disease MR	0.0925	1.549	0.930	2.581
Non ischemic cardiomyopathy	0.6138	1.124	0.713	1.771

*sPAP* systolic pulmonary artery pressure, *MR* mitral regurgitation, *AF* atrial fibrillation, *CRT* cardiac resynchronization therapy, *MR* mitral regurgitation, *TOE* transesophageal echocardiography. *p < 0.05

### 1-year mortality according to 3D-VCA

**Fig. 3:**
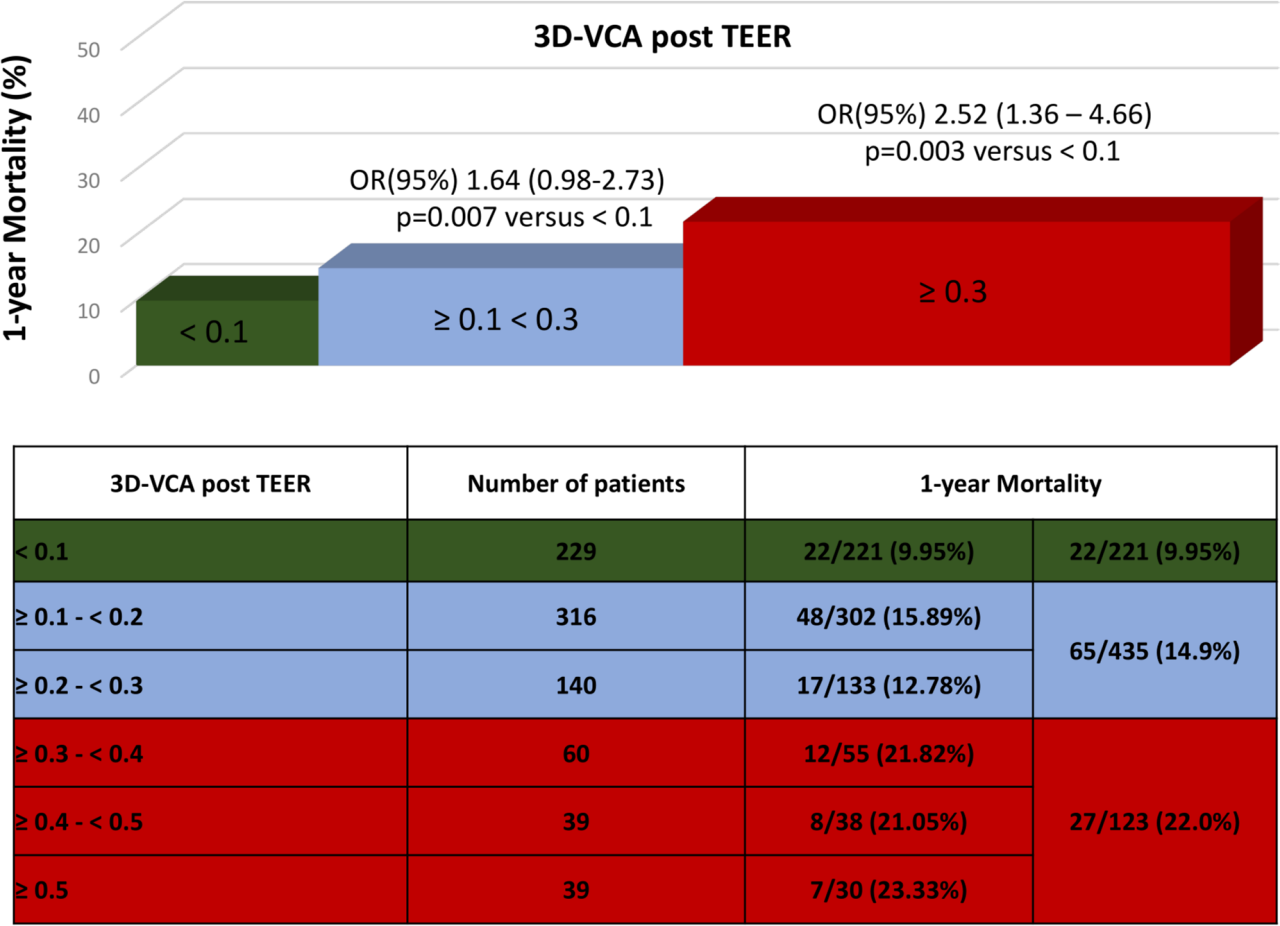
Association of 3D-VCA and 1-year mortality. 3D-VCA assessing residual MR following transcatheter mitral valve repair (TEER) related to 1-year mortality. Bar graphs indicate grouped 3D-VCA post TEER (< 0.1 cm^2^, ≥ 0.1 < 0.3 cm^2^ and ≥ 0.3 cm^2^). *OR* odds ratio

1-year clinical follow-up was completed in 779 patients (94.7%) of the total cohort (3D-VCA < 0.1 cm^2^: 221 patients, 96.5%; 3D-VCA ≥ 0.1 < 0.3 cm^2^: 435 patients, 95.4%; 3D-VCA ≥ 0.3 cm^2^: 123 patients, 89.1%). Notably, patients with trace or mild residual MR showed significantly lower 1-year mortality rates. Specifically, the 1-year mortality rates were 10.5% in the 3D-VCA < 0.1 cm^2^ group, 16.0% in the 3D-VCA ≥ 0.1 < 0.3 cm^2^ group, and 24.8% in the 3D-VCA ≥ 0.3 cm^2^ group (Fig. 2). A higher 3D-VCA measurement post-mitral TEER was associated with increased 1-year mortality, as shown in Fig. 3.

An analysis with the 3 predefined 3D-VCA groups revealed that group ≥ 0.1 < 0.3 cm^2^ is significantly different from the 3D-VCA < 0.1 group (p = 0.01) and 3D-VCA ≥ 0.3 group (p < 0.001) demonstrating increasing 1-year mortality over these groups (Fig. 3). Furthermore, re-intervention or mitral valve surgery was more frequently required in the 3D-VCA ≥ 0.3 cm^2^ group (3D-VCA < 0.1 cm^2^: 1.1%; 3D-VCA ≥ 0.1 < 0.3 cm^2^: 4.4%; and 3D-VCA ≥ 0.3 cm^2^: 8.8%; p = 0.016).

### Comparison of 3D-VCA to LAP and 2D-TOE

Patients were categorized based on MR severity: MR 0/I (none, trace, mild MR), MR II (moderate MR), or MR III (severe MR), as determined by post-procedural TOE following mitral TEER. We observed a 1-year mortality rate of 15.9% for MR 0/I, 22.4% for MR II, and 25.0% for MR III, as assessed by post-procedural 2D-TOE, indicating a trend without a significant difference (log-rank p-value: 0.063). Similarly, we conducted an analysis based on LAP post-TEER, categorizing patients into LAP < 20 mmHg, LAP 20–35 mmHg, and LAP > 35 mmHg. The 1-year mortality rates were 16.2% for LAP < 20 mmHg, 16.3% for LAP 20–35 mmHg, and 25.0% for LAP > 35 mmHg, again showing a trend without a significant difference (log-rank p-value: 0.064). A c-statistic for predicting 1-year mortality was performed: 3D-VCA: 0.6 (CI: 0.52–0.63), 2D-TOE: 0.5 (CI: 0.43–0.58) and LAP:0.5 (CI: 0.43–0.56).

### Interobserver variability

To estimate interobserver variability corelab 3D-VCA data of 47 patients were compared to the corresponding 3D-VCA assessed by the investigators. Pearson correlation coefficient (PCC) and Bland Altman Analysis of 3D-VCA (investigator versus corelab) demonstrated good reliability (PCC: 0.86; p < 0.001; supp. Figure 1).

### 12-months clinical and echocardiographic follow-up

In the MITRA-PRO cohort, mitral TEER reduced 3D-VCA significantly compared to baseline. In transthoracic echocardiography, MR grading improved at discharge compared to baseline across all groups, with significantly better results in patients with a 3D-VCA < 0.1 cm^2^ (Supplemental Table 3). However, at 12-month follow-up, the number of patients who underwent transthoracic echocardiography at individual study centers was low (3D-VCA < 0.1 cm^2^: 38; 3D-VCA ≥ 0.1 < 0.3 cm^2^: 83; 3D-VCA ≥ 0.3 cm^2^: 33), leading to no statistical difference in MR grading between the three 3D-VCA groups (supplemental Table 3).

**Fig. 4:**
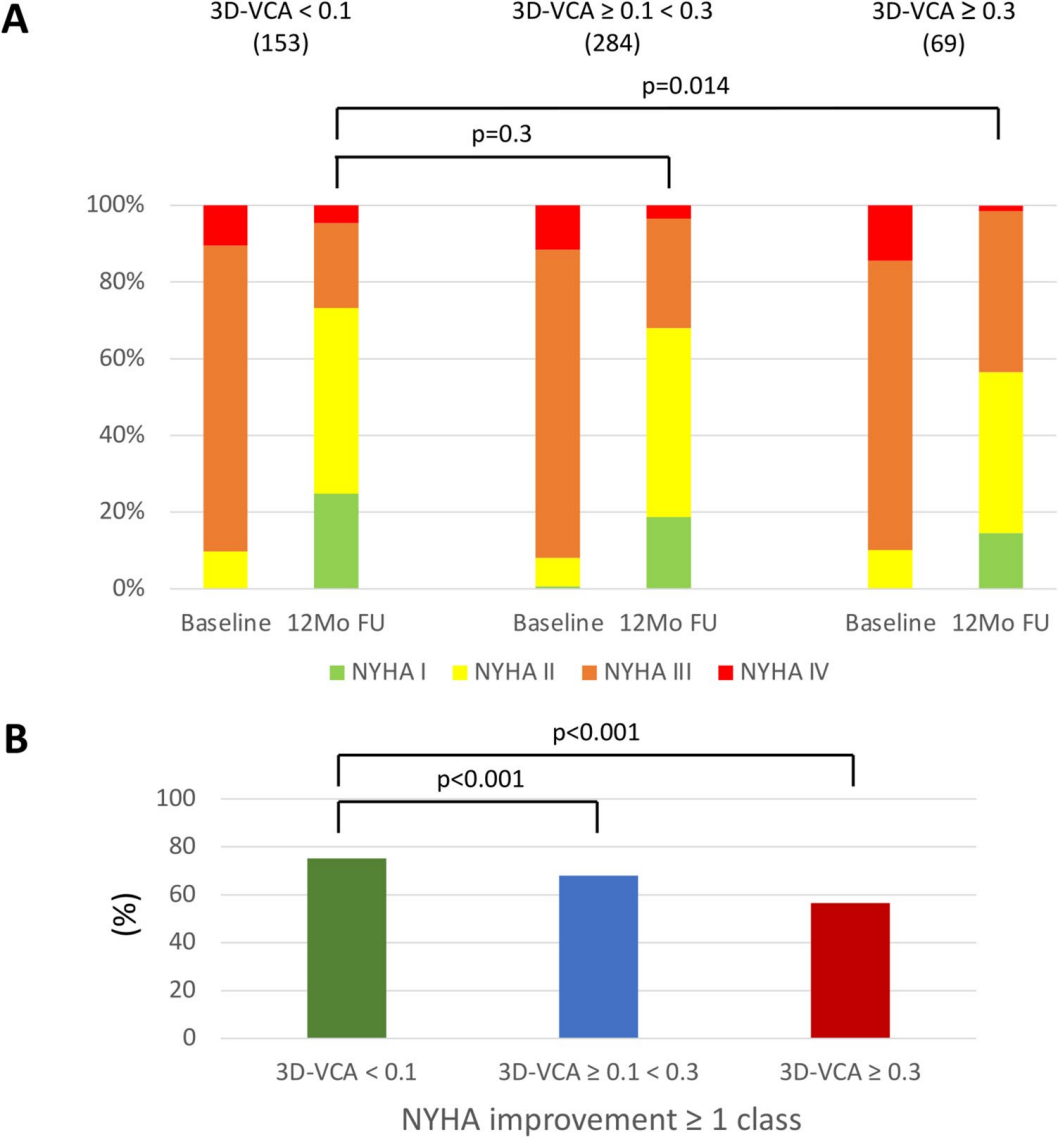
NYHA classification according to 3D-VCA groups. **A** Distribution of NYHA classification at baseline, discharge and 12-months follow-up according to 3D-VCA groups (< 0.1 cm^2^, ≥ 0.1 < 0.3 cm^2^ and ≥ 0.3 cm^2^). **B** NYHA improvement ≥ 1 class according to 3D-VCA groups (< 0.1 cm^2^, ≥ 0.1 < 0.3 cm^2^ and ≥ 0.3 cm^2^). *3D-VCA* 3D-Vena Contracta Area

Furthermore, NYHA classification was improved at 12-months follow-up after mitral TEER (Fig. 4A). Baseline NYHA classification was comparable in all 3D-VCA groups, while in 3D-VCA < 0.1 cm^2^ the number of patients with NYHA I/II at 12-months follow-up was significantly higher compared to 3D-VCA ≥ 0.3 cm^2^ indicating an association of residual MR and improvement of symptoms (Fig. 4A). Also, improvement in NYHA classification (at least one class) at 12-months follow-up following mitral TEER was significantly higher in 3D-VCA < 0.1 cm^2^ compared to 3D-VCA ≥ 0.3 cm^2^ (Fig. 4B), which further strengthens the hypothesis, that residual MR after mitral TEER is directly linked to symptomatic improvement.

## Discussion

The MITRA-PRO registry evaluated the impact of intraprocedural residual mitral regurgitation on clinical outcomes after mitral TEER and revealed in patients with mild residual MR a significantly lower mortality and rehospitalization rate at one year after TEER [1]. No difference between degenerative (primary), functional (secondary) or mixed MR was observed [1]. However, in a real-world setting interventional cardiologists seek for a simple and straight-forward approach of evaluating residual MR following mitral TEER. Color Doppler echocardiography is able to allow rapid regurgitation assessment, but has limitations [13]. Especially in the context of multiple residual MR jets post TEER color Doppler echocardiography might lead to over- or underestimation of residual MR [14]. Furthermore, effective regurgitant orifice area calculation based on the PISA method is not recommended for flow quantification especially in case of multiple residual regurgitation jets [13]. Finally, the classical multi-parametric MR assessment by TTE is not feasible during the interventional procedure. The c-statistic of 0.5 for 2D-TOE assessed in this MITRA-PRO analysis highlights its limitations in post mitral-TEER assessment, and reinforces the additional usage of 3D techniques for residual MR quantification in this context. Therefore, 3D-VCA is an emerging parameter for residual MR evaluation following mitral TEER [2–4]. However, no validated grading of 3D-VCA exists and today current guidelines do not recommend 3D-VCA for residual MR gradation [5, 6]. This study confirmed that intraprocedural 3D-VCA assessment by experienced interventional echocardiographers of residual MR alone with standardized hemodynamic conditions is able to predict 1-year mortality. Therefore, a 3D-VCA of < 0.07 cm^2^ showed the lowest 1-year mortality, while a 3D-VCA ≥ 0.3 cm^2^ is associated with the poorest outcome. Also, improvement of NYHA classification at 12-months follow-up was associated with a lower 3D-VCA post mitral TEER. Avenatti et al. showed a 3D-VCA threshold of 0.27 cm^2^ to be indicative for moderate residual MR, which is confirmed by our findings of excess mortality in patients with a 3D-VCA ≥ 0.3 cm^2^ [3]. However, we showed that also a 3D-VCA ≥ 0.07 cm^2^ is associated with increased 1-year mortality compared to less residual MR post mitral TEER.

3D-VCA assessment has been reported feasible and reproducible for both eccentric and multiple regurgitation jets, which are characteristic findings after mitral TEER [12, 14, 15]. Furthermore, in-vitro validation and cardiac MRI comparison studies showed promising results for mitral regurgitation quantification using 3D-VCA [16–19]. Moreover, a low interobserver variability was reported for both baseline and post-TEER 3D-VCA assessment [3]. In the MITRA-PRO registry 3D-VCA was used in a real-world scenario of a large cohort of patients with primary, secondary or mixed MR. Of note, experience in this technique was a requirement for usage in the study protocol. MITRA-PRO was a nonrandomized registry and intraprocedural evaluation of MR was done at the end of mitral TEER using a web-based system, which excluded post-hoc changes of the MR assessment. Clinical follow-ups were done investigator independent by a central site excluding investigator bias. In this real-world setting 3D-VCA proofed feasibility and predicted 1-year mortality, while lowest 1-year mortality was found for 3D-VCA < 0.1 cm^2^. Severe MR was defined as a 3D-VCA of > 0.4 cm^2^ and moderate MR as 3D-VCA of > 0.3 cm^2^ in previous studies [3]. In MITRA-PRO a 3D-VCA ≥ 0.3 revealed the highest mortality and showed reduced clinical improvement following mitral TEER.

A recent study demonstrated the value of real-time monitoring of LAP during TEER to predict clinical outcomes. An increase in mean LAP was associated with worse clinical outcomes at short-term follow-up [20]. Therefore, we analyzed the impact of LAP assessed after deployment of the last TEER device on 1-year mortality. No significant difference between 3 predefined LAP groups and a c-statistic of 0.5 for 1-year mortality were found. Furthermore, an analysis of 1-year mortality for residual mitral regurgitation class 0/I (none, trace, mild MR), class II (moderate) and III (severe) assessed by intraprocedural 2D-TOE post TEER was performed. The predictive performance regarding 1-year mortality was similar between 2D-TOE and LAP (both with a c-statistic of 0.5). A c-statistic revealed 0.6 for 3D-VCA, which indicate that 3D-VCA has modest predictive power and should not be used in isolation for clinical prognosis assessment. Furthermore, the overlapping confidence intervals of 3D-VCA, LAP and 2D-TOE indicate no superiority of a single parameter in the assessment of long-term survival. Predicting 1-year mortality after mitral TEER is complex, involving multiple factors beyond residual MR severity, such as renal function or ventricular function. An optimal approach would involve integrating 3D-VCA with other modalities, such as comprehensive 2D echocardiographic assessments, clinical parameters, and hemodynamic data such as LAP, to provide a robust evaluation of patient outcomes post-TEER.

Avenatti et al. documented good interobserver variability for baseline 3D-VCA (ICC = 0.84) and excellent variability for post-procedural 3D-VCA (ICC = 0.93) [3]. Our findings align with these results through a Bland–Altman Analysis and Pearson's Correlation Coefficient comparing 3D-VCA values obtained by investigators and an independent core lab. However, our analysis was not designed to investigate interobserver variability and is constrained by the limited number of patients with core lab 3D-VCA assessment due to missing raw 3D-VCA data. Also, 3D-VCA is an echocardiographic parameter that can be influenced by image quality and the investigator’s experience, factors that may contribute to interobserver variability.

This analysis of the MITRA-PRO registry demonstrated the impact of residual MR assessed by 3D-VCA following TEER on 1-year mortality. Predictors of a residual MR with a 3D-VCA ≥ 0.3 cm^2^ were permanent atrial fibrillation, severe baseline MR and increased sPAP per 20 mmHg. A significant residual MR (3D-VCA ≥ 0.3) was associated with more severe MR grading at baseline, higher baseline sPAP, a higher proportion of mixed MR cases, the use of multiple TEER devices, and extended procedure times, all of which indicate more complex technical cases. Complex TEER cases lead to suboptimal results, as indicated by residual MR, which is directly linked to increased mortality. This critical aspect needs careful consideration in patient selection before mitral TEER.

In summary, an optimal procedural result is beneficial for long-term survival after TEER. Procedural 3D-VCA alone is a valuable parameter to quantify residual MR following mitral TEER and it is associated with 1-year mortality in a setup of experienced interventional echocardiographers.

## Limitations

Assessment of residual MR was done by MITRA-PRO investigators and investigator bias cannot be excluded. Also, 3D-VCA was only assessed in a limited number of patients by the MITRA-PRO corelab due to missing 3D-VCA raw data. However, our results on interobserver variability were supported by a recent publication with similar findings (3). Furthermore, in this real-world, all-comer registry guideline directed medical treatment (GDMT) was not controlled at 1-year follow-up. Therefore, changes in GDMT might have influenced the outcome results. Also, accuracy of 3D-VCA tracing can be affected by non-standardized settings of different ultrasound machine manufacturers, making generalization of cutoff values difficult. MITRA-PRO started enrolling patients in 2016 and modern rendering tools were not available, which could increase accuracy of 3D-VCA assessment. Residual MR jets follow different trajectories and identification of the true perpendicular plane for each jet is necessary. Transthoracic echocardiography at the 12-month follow-up was conducted by individual study centers. However, the number of echocardiograms performed at 12 months was low. Due to this loss to follow-up of patients, no valid conclusions can be drawn about the durability of mitral TEER in the MITRA-PRO study.

## Conclusion

These results of a MITRA-PRO registry demonstrated the feasibility of 3D-VCA assessment for quantification of residual MR following mitral TEER. Furthermore, residual MR assessed by 3D-VCA is associated with postprocedural 1-year mortality in a prospective large scale multicenter trial. A 3D-VCA ≥ 0.07 cm^2^ is associated with increased 1-year mortality, a higher rate of reintervention and reduced symptomatic improvement.

## Supplementary Information

Below is the link to the electronic supplementary material.

Supplementary file1 (TIFF 398 KB)

Supplementary file2 (DOCX 36 KB)

Supplementary file3 (DOCX 16 KB)

Supplementary file4 (DOCX 15 KB)

## Data Availability

The data that support the findings of this study are available from the corresponding author upon reasonable request.
