# Supplementary figures and images for: Intraprocedural 3D-vena contracta area predicts survival after transcatheter edge-to-edge repair: results from MITRA-PRO registry
Source: Clin Res Cardiol. 2024 Dec 9;114(7):867–77. doi: 10.1007/s00392-024-02580-6 (PMC12202621; doi:10.1007/s00392-024-02580-6)

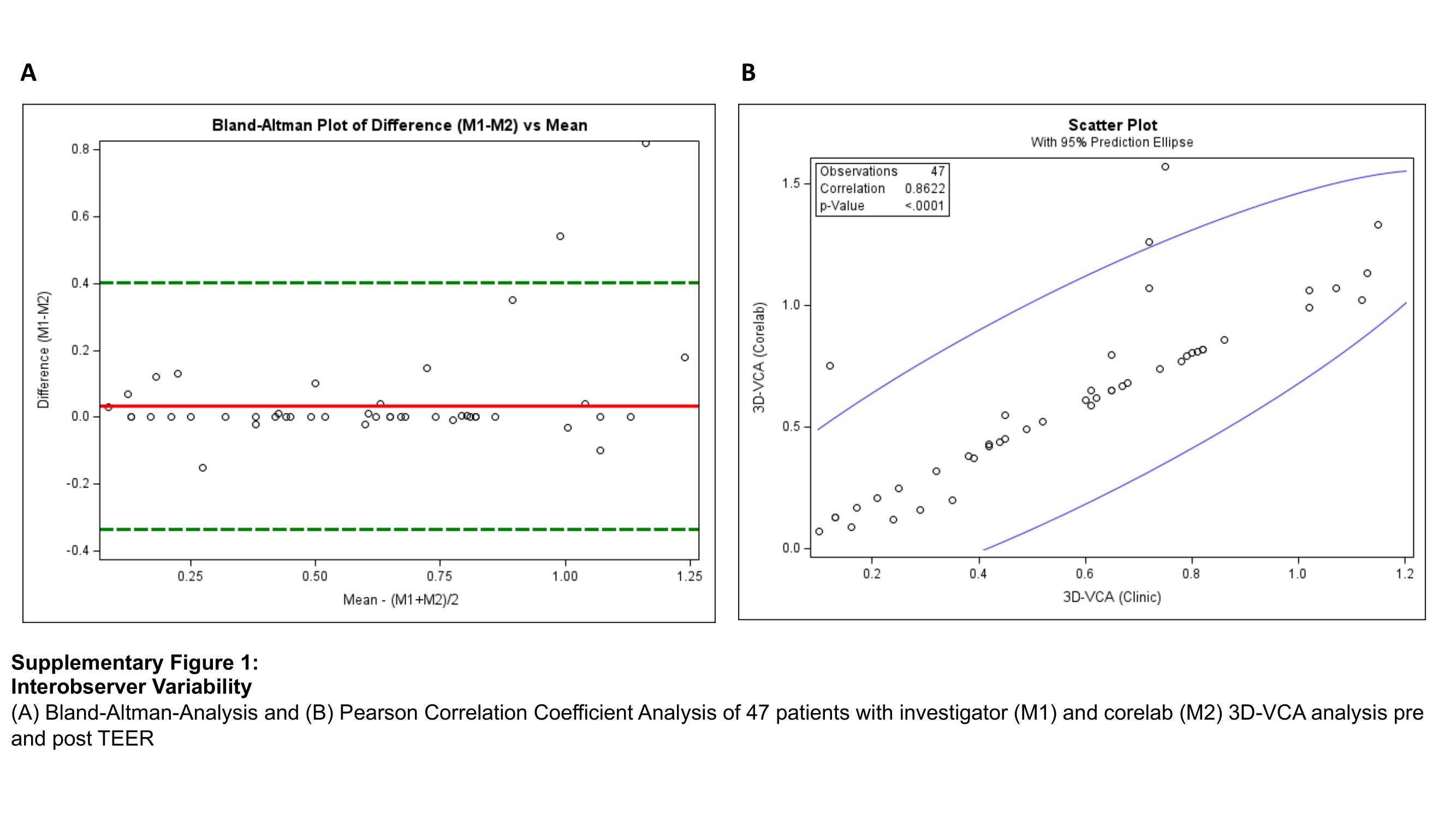

Supplement: Supplementary file 1 — Supplementary file1 (TIFF 398 KB) [file 392_2024_2580_MOESM1_ESM.tiff]
