# Supplementary material for: Intraprocedural 3D-vena contracta area predicts survival after transcatheter edge-to-edge repair: results from MITRA-PRO registry
Source: Clin Res Cardiol. 2024 Dec 9;114(7):867–77. doi: 10.1007/s00392-024-02580-6 (PMC12202621; doi:10.1007/s00392-024-02580-6)
Supplement: Supplementary file 2 — Supplementary file2 (DOCX 36 KB) [file 392_2024_2580_MOESM2_ESM.docx]

| **MITRA-PRO study center** | **Echocardiography system and 3D analysis software** | **Number of patients**  **with 3D-VCA** |
| --- | --- | --- |
| Department of Cardiology, Helios Klinikum Siegburg, Siegburg, Germany | Philips iE33/EPIQ 7  QLAB | 57 |
| Department of Cardiology, Heart Centre Niederrhein, Helios Clinic Krefeld, Krefeld, Germany | GE Vivid E9/E95  EchoPAC | 91 |
| Department of Cardiology, Klinikum der Universität München, Ludwig-Maximilians-Universität, Munich, Germany | Philips iE33/EPIQ 7  QLAB | 78 |
| Department of Cardiology, Asklepios Klinik St. Georg, Hamburg | Philips iE33/EPIQ 7  QLAB | 67 |
| Department of Internal Medicine III, HELIOS Klinikum Erfurt, Erfurt, Germany | Philips iE33/EPIQ 7  QLAB | 6 |
| Department of Cardiology and Angiology, Justus-Liebig-University, Giessen, Germany | Philips iE33/EPIQ 7  QLAB | 10 |
| Department of Internal Medicine and Cardiology, Krankenhaus der Barmherzigen Brüder, Trier, Germany | Philips iE33/EPIQ 7  QLAB | 30 |
| Department of Cardiology, Elisabeth Krankenhaus, Essen, Germany | GE Vivid E9/E95  EchoPAC | 129 |
| Department of Cardiology and Angiology II, University Heart Center Freiburg, Bad Krozingen, Germany | Philips iE33/EPIQ 7  QLAB | 109 |
| Department of Cardiology, Heart Center University of Cologne, Cologne, Germany | Philips iE33/EPIQ 7  QLAB | 55 |
| Department of Cardiology, Unfallkrankenhaus Berlin, Berlin, Germany | Philips iE33/EPIQ 7  QLAB | 31 |
| Heart Valve Center, Universitätsmedizin Mainz, Johannes Gutenberg-University Mainz, Germany | Philips iE33/EPIQ 7  QLAB | 9 |
| Department of Cardiology, Cardioangiologisches Centrum Bethanien (CCB), Frankfurt, Germany | GE Vivid E9/E95  EchoPAC | 50 |
| Department of Cardiology, Heart Center Leipzig, University of Leipzig, Germany | Philips iE33/EPIQ 7  QLAB | 5 |
| Department of Cardiology and Cardiovascular Medicine, University Hospital Tuebingen, Tuebingen, Germany | Philips iE33/EPIQ 7  QLAB | 37 |
| Department of Cardiology, University Hospital Würzburg, Würzburg, Germany | GE Vivid E9/E95  EchoPAC | 10 |
| Department of Cardiology, Katholisches Marienkrankenhaus Hamburg, Hamburg, Germany | Philips iE33/EPIQ 7  QLAB | 46 |
| Department of Cardiology, Herz- und Diabeteszentrum NRW, Bad Oeynhausen, Germany | GE Vivid E9/E95  EchoPAC | 11 |
| Total patients |  | 831 |
| Patients with Philips / QLAB 3D-VCA analysis |  | 540 |
| Patients with GE / EchoPAC 3D-VCA analysis |  | 291 |

Supplementary table 1: Study centers, number of patients enrolled and echocardiography system used for 3D-VCA calculation.
