# Supplementary material for: Intraprocedural 3D-vena contracta area predicts survival after transcatheter edge-to-edge repair: results from MITRA-PRO registry
Source: Clin Res Cardiol. 2024 Dec 9;114(7):867–77. doi: 10.1007/s00392-024-02580-6 (PMC12202621; doi:10.1007/s00392-024-02580-6)
Supplement: Supplementary file 3 — Supplementary file3 (DOCX 16 KB) [file 392_2024_2580_MOESM3_ESM.docx]

|  | **3D VCA < 0.1 cm^2^**  **n = 229** | | **3D VCA ≥ 0.1 < 0.3 cm^2^**  **n = 456** | | **3D VCA ≥ 0.3 cm^2^**  **n = 138** | | **p-value** |
| --- | --- | --- | --- | --- | --- | --- | --- |
|  | **n** | **% or Mean ± SD or Median (Min,Max)** | **n** | **% or Mean ± SD or Median (Min,Max)** | **n** | **% or Mean ± SD or Median (Min,Max)** |  |
| TEER devices | 229 | 1.5 ± 0.8 | 456 | 1.6 ± 0.6 | 138 | 1.8 ± 0.8 | <0.001 |
| Procedure time (min) | 229 | 74.0 (52.0, 91.0) | 456 | 73.0 (56.0, 100.0) | 138 | 90.0 (69.0, 128.0) | <0.001 |
| **Hemodynamics pre TEER** |  |  |  |  |  |  |  |
| BP systolic (mmHg) | 229 | 119.0 ± 20.3 | 456 | 117.7 ± 19.4 | 138 | 114.8 ± 17.5 | 0.22 |
| BP diastolic (mmHg) | 229 | 59.8 ± 12.5 | 456 | 61.1 ± 13.1 | 138 | 61.1 ± 11.3 | 0.48 |
| CVP (mmHg) | 229 | 10.1 ± 5.3 | 456 | 11.8 ± 5.9 | 138 | 13.6 ± 6.7 | <0.001 |
| LVEDP (mmHg) | 229 | 13.3 ± 6.0 | 456 | 14.9 ± 6.9 | 138 | 15.2 ± 6.5 | 0.12 |
| LA pressure (mmHg) | 229 | 29.0 ± 13.8 | 456 | 31.1 ± 13.8 | 138 | 33.8 ± 15.1 | 0.007 |
| **Hemodynamics post TEER** |  |  |  |  |  |  |  |
| BP systolic (mmHg) | 229 | 120.1 ± 17.9 | 456 | 120.9 ± 50.1 | 138 | 116.6 ± 16.3 | 0.18 |
| BP diastolic (mmHg) | 229 | 58.8 ± 12.8 | 456 | 60.7 ± 13.7 | 138 | 60.0 ± 12.2 | 0.18 |
| CVP (mmHg) | 229 | 10.5 ± 5.2 | 456 | 12.0 ± 5.6 | 138 | 12.6 ± 6.3 | 0.006 |
| LVEDP (mmHg) | 229 | 13.0 ± 9.4 | 456 | 13.2 ± 6.1 | 138 | 14.1 ± 7.3 | 0.43 |
| LA pressure (mmHg) | 229 | 19.3 ± 8.4 | 456 | 20.5 ± 9.1 | 138 | 23.3 ± 10.3 | <0.001 |
| **Complications** |  |  |  |  |  |  |  |
| Stroke | 1 | 0.4 | 2 | 0.4 | 1 | 0.8 | 0.89 |
| Myocardial infarction | 0 | 0.0 | 0 | 0.0 | 0 | 0.0 |  |
| Pericardial tamponade | 1 | 0.4 | 3 | 0.7 | 0 | 0.0 | 0.62 |
| Renal failure | 19 | 8.3 | 37 | 8.1 | 20 | 14.7 | 0.056 |
| Dialysis | 4 | 1.7 | 15 | 3.3 | 2 | 1.5 | 0.33 |
| Major Bleeding | 7 | 3.1 | 27 | 5.9 | 10 | 7.4 | 0.15 |
| Re-Intervention / Operation | 0 | 0.0 | 5 | 1.1 | 1 | 0.8 | 0.28 |
| **In-hospital clinical course** |  |  |  |  |  |  |  |
| In-hospital mortality | 3 | 1.3 | 9 | 2.0 | 4 | 2.9 | 0.56 |
| MACCE (Death, Stroke, MI) | 4 | 1.8 | 9 | 2.0 | 5 | 3.8 | 0.39 |
| MACE (Death, MI) | 3 | 1.3 | 9 | 2.0 | 4 | 3.1 | 0.52 |

**Supplementary table 2. Procedural parameters and clinical course following mitral TEER.**

BP=blood pressure; TEER=transcatheter edge-to-edge repair; LVEDP=left ventricular endiastolic pressure; LA=left atrial; MACCE=major adverse cardiac and cerebrovascular events; MACE= major adverse cardiac events; MI=myocardial infarction. Major bleeding is defined as any bleeding requiring transfusion.
