# Supplementary material for: Intraprocedural 3D-vena contracta area predicts survival after transcatheter edge-to-edge repair: results from MITRA-PRO registry
Source: Clin Res Cardiol. 2024 Dec 9;114(7):867–77. doi: 10.1007/s00392-024-02580-6 (PMC12202621; doi:10.1007/s00392-024-02580-6)
Supplement: Supplementary file 4 — Supplementary file4 (DOCX 15 KB) [file 392_2024_2580_MOESM4_ESM.docx]

|  | **3D VCA < 0.1 cm^2^**  **n = 224** | | **3D VCA ≥ 0.1 < 0.3 cm^2^**  **n = 451** | | **3D VCA ≥ 0.3 cm^2^**  **n = 135** | | **p-value** |
| --- | --- | --- | --- | --- | --- | --- | --- |
| **MR grading baseline** |  |  |  |  |  |  |  |
| None |  | 0.0% (0/224) |  | 0.0% (0/451) |  | 0.0% (0/135) | <0.001 |
| Mild |  | 0.9% (2/224) |  | 0.4% (2/451) |  | 0.0% (0/135) |  |
| Moderate |  | 16.1% (36/224) |  | 11.3% (51/451) |  | 3.7% (5/135) |  |
| Severe |  | 83.0% (186/224) |  | 88.2% (398/451) |  | 96.3% (130/135) |  |
| **MR grading discharge** |  |  |  |  |  |  |  |
| None |  | 10.7% (24/224) |  | 2.4% (11/451) |  | 0.7% (1/135) | <0.001 |
| Mild |  | 74.1% (166/224) |  | 73.2% (330/451) |  | 51.1% (69/135) |  |
| Moderate |  | 13.8% (31/224) |  | 21.7% (96/451) |  | 36.3% (49/135) |  |
| Severe |  | 1.3% (3/224) |  | 2.7% (12/451) |  | 11.9% (16/135) |  |

|  | **3D VCA < 0.1 cm^2^**  **n = 38** | | **3D VCA ≥ 0.1 < 0.3 cm^2^**  **n = 83** | | **3D VCA ≥ 0.3 cm^2^**  **n = 33** | | **p-value** |
| --- | --- | --- | --- | --- | --- | --- | --- |
| **MR grading baseline** |  |  |  |  |  |  |  |
| None |  | 0.0% (0/38) |  | 0.0% (0/83) |  | 0.0% (0/33) | 0.14 |
| Mild |  | 0.0% (0/38) |  | 0.0% (0/83) |  | 0.0% (0/33) |  |
| Moderate |  | 13.2% (5/38) |  | 16.9% (14/83) |  | 3.0% (1/33) |  |
| Severe |  | 86.8% (33/38) |  | 83.1% (69/83) |  | 97.0% (32/33) |  |
| **MR grading 12-months FU** |  |  |  |  |  |  |  |
| None |  | 7.9% (3/38) |  | 7.2% (6/83) |  | 12.1% (4/33) | 0.50 |
| Mild |  | 71.1% (27/38) |  | 57.8% (48/83) |  | 60.6% (20/33) |  |
| Moderate |  | 15.8% (6/38) |  | 33.7% (28/83) |  | 12.1% (4/33) |  |
| Severe |  | 5.3% (2/38) |  | 1.2% (1/83) |  | 15.2% (5/33) |  |

**Supplementary table 3. Mitral regurgitation grading at baseline, discharge and 12-months follow-up.** MR=mitral regurgitation; FU=follow-up.
